# A Simple Method for Anchoring Silver and Copper Nanoparticles on Single Wall Carbon Nanotubes

**DOI:** 10.3390/nano9101416

**Published:** 2019-10-04

**Authors:** Mariana M. Silva, Daniel Ribeiro, Eunice Cunha, M. Fernanda Proença, Robert J. Young, Maria C. Paiva

**Affiliations:** 1 Institute for Polymers and Composites, University of Minho—Campus of Azurém, 4800-058 Guimarães, Portugal; b8523@dep.uminho.pt (M.M.S.); daniel.ribeiro@dep.uminho.pt (D.R.); 2 National Graphene Institute and School of Materials, University of Manchester, Manchester M13 9PL, UK; eunice.cunha-2@manchester.ac.uk (E.C.);; 3 Centre of Chemistry, University of Minho—Campus of Gualtar, 4710-057 Braga, Portugal; fproenca@quimica.uminho.pt

**Keywords:** 1,3-dipolar cycloaddition, functionalization of SWCNT, metal anchoring

## Abstract

Single walled carbon nanotubes (SWCNT) were functionalized using the 1,3-dipolar cycloaddition reaction of an azomethine ylide under solvent-free conditions, a one-pot procedure that yields pyrrolidine type of groups at the nanotubes surface. The functionalized SWCNT were further decorated with Ag and Cu nanoparticles by reduction of the corresponding metal salts in dimethylformamide. The extensive reduction of silver from its nitrate was observed, as well as the partial reduction of copper from its acetate. X-ray photoelectron spectroscopy (XPS) confirmed the functionalization of SWCNT with pyrrolidine that provided anchoring sites for the metal nanoparticles. Metal nanoparticles (NP) were formed at the surface of the organically functionalized SWCNT in higher yields as compared to the same procedure carried out with pristine SWCNT. This was observed using scanning electron microscopy (SEM) and quantified by XPS. Raman spectroscopy demonstrated that functionalization and metal decoration of the SWCNT did not induce structural damage to the SWCNT.

## 1. Introduction

Outstanding properties have been attributed to single walled carbon nanotubes (SWCNT) [1,2,3,4,5,6,7,8,9,10,11] since their discovery by Iijima [12]. The chemical inertia of carbon nanotubes (CNT), however, limits their application and much work has been undertaken in the field of non-covalent and covalent functionalization in attempts to increase their solubility or improve their interfacial bonding with different matrix materials. The underlying inertia comes from the tight sp^2^ bonds of the hexagonal carbon lattice as well as the stable tubular structure and strong van der Waals interactions that hold the tubes together in bundles. The reactivity of SWCNT is higher at the end caps due the curvature of the C-C network, at Stone-Wales defects [13], and for smaller diameter SWCNT, also due to curvature effects [14]. Noncovalent and covalent modifications as well as decoration with metal nanoparticles are strategies that were applied to fulfil the purpose of increased solubility, for the exploitation of the SWCNT properties and their reactivity, to develop hybrid materials for several applications [15,16].

Georgakilas et al. [17] described the 1,3-dipolar cycloaddition (DCA) of azomethine ylides to the surface of SWCNT, a reaction adapted from previous studies with fullerenes [18]. Since then a series of 1,3-dipolar cycloadditions of azomethine ylides to SWCNT has been reported [17,19,20,21,22,23,24,25,26,27] using Prato’s reaction route as well as microwave assisted synthesis. DCA reactions are expected to be less damaging for CNT than fluorination and oxidation reactions, thus minimizing the defects inflicted by functionalization while enhancing the reactivity of the CNT surface. Nevertheless, these reactions can be time consuming (several days, when carried out in solution) and are hardly scalable. Paiva et al. reported the DCA of azomethine ylides to the surface of multi wall CNT (MWCNT) [28] and carbon nanofibers [29] through a simple and scalable one-pot solvent free method.

Frequently, the cycloaddition reactions reported in the literature for SWCNT require prior purification [19,21,22,23,25], since commercially available SWCNT contain impurities such as amorphous carbon and metal catalysts that may affect the chemical reactions. The reagents used to modify the surface of SWCNT can functionalize other carbonaceous materials present, as well as complex with residual catalyst, thus reducing the extent of surface functionalization of the CNT.

The reduction of Ag and Cu salts to their elemental form has been studied and was successfully achieved using different approaches, such as described in the literature for the production of metal decorated SWCNT, with Ag [30,31,32,33,34,35] and Cu [36,37]. Dimethylformamide (DMF) is commonly used as a solvent, however this amide may be used as a reducing agent for some metal salts. The preparation of Ag nanoparticles (NP) by reduction of AgNO_3_ in DMF is described in the literature [38] and has been used as a route for decorating MWCNT [39,40] and graphene oxide [41], however with limited efficiency in the absence of anchoring sites along the CNT surface. Sulfur and amine groups attached to the side wall of CNT were observed to be potential stabilizers of cations, as previously reported by Ma et al. [40], acting as anchoring sites for Ag NP.

Hybrid CNT/metal NP are of interest in applications such as sensing [30,36,42,43], energy storage [31], health monitoring [44], supercapacitors [45] as well as transistors and memory devices [46]. However, in spite of the excellent properties of SWCNT, their effective transfer to the final application is hindered by the presence of SWCNT bundles, structural defects, and conductivity variation with nanotube chirality, etc. Moreover, their contact resistance with metals poses a potential problem to device performance, and this has been the focus of several studies [47,48]. The modification of the inter-nanotube bundles by metal nanoparticle decoration to produce hybrid networks has shown potential for the reduction of contact resistance between the nanotubes [49], which is interesting for the above-mentioned applications. Another potential application for hybrid SWCNT/metal NP is the preparation of substrates for surface-enhanced Raman scattering (SERS) [50,51]. It was demonstrated that the SWCNT Raman response can be considerably enhanced by the attachment of metallic NP [50]. Attaching Ag NP to the surface of SWCNT was also used for the assembly of active surfaces for a label-free detection of ssDNA [30] and Cu NP were applied for enhancing the sensitivity of a H_2_S sensor [36].

In the present study we report the organic functionalization of SWCNT using the solvent-free 1,3-dipolar cycloaddition of an azomethine ylide, adapted from the MWCNT functionalization method proposed by Paiva et al. [29]. The outcome of the functionalization on pristine and purified SWCNT was evaluated. Furthermore, the functionalized SWCNT (f-SWCNT) were decorated with Ag and Cu nanoparticles through the reduction of AgNO_3_ and Cu(CH_3_COO)_2_ in DMF in the presence of suspended f-SWCNT, yielding hybrid SWCNT (h-SWCNT). The functionalized and hybrid materials were analyzed by X-ray photoelectron spectroscopy (XPS), Raman spectroscopy, thermogravimetric analysis (TGA), and scanning electron microscopy with energy dispersive spectroscopy (EDS) analysis, demonstrating the effectiveness of this simple methodology to synthetize h-SWCNT.

## 2. Materials and Methods

### 2.1. Purification of the SWCNT

SWCNT Tuball™ were purchased from OCSiAl Ltd. (Luxembourg, Batch 109-16092015). The chemical purification process was adapted from the procedures proposed by Wang et al. [52] and Clancy et al. [53]. SWCNT powder (25 mg) was stirred in a mixture of HCl (1 M, 25 mL) and H_2_O_2_ (30% wt/wt, 25 mL), from Fisher Scientific (UK), and heated at 60 °C. After each hour, 25 mL of both reactants were added for a total of 4 h. Vacuum filtration was carried out with a Nylon membrane (GE Healthcare, IL, USA, Whatman™, 0.45 µm pore size) and the filtrate was washed with deionized water until the pH of the filtration liquid reached seven.

### 2.2. Functionalization of Pristine and Purified SWCNT

The functionalization procedure of the SWCNT was carried out using the solvent-free DCA reaction. The alfa amino acid and aldehyde used were N-benzyloxycarbonylglycine (Z-Gly-OH) and paraformaldehyde (PFA), respectively (both acquired from ACROS Organics, Belgium, with >96% purity). The procedure was adapted from that described in the literature for nanofibers [28] and MWCNT [29]. This procedure was adjusted for the functionalization of SWCNT considering their larger surface area and higher curvature compared to MWCNT. Two different ratios of SWCNT to Z-Gly-OH were tested. The reaction conditions employed are described in Table 1.

The reaction products were filtered through a qualitative filter, 25–30 µm pore size (Prat Dumas, France), and washed with a sequence of solvents, namely hexane, ethanol, and acetone (from Fisher Scientific, UK). The functionalized pristine SWCNT (f-S1 and f-S6, with ratios of 1:1 and 1:6 of SWCNT to Z-Gly-OH, respectively) were dried at 150 °C for 1 h under vacuum and the purified SWCNT were dried at 200 °C (f-SP1). A buckypaper-like structure (tightly packed SWCNT bundles) was obtained for all samples.

### 2.3. Decoration of SWCNT with Metal Salts

Decoration with Ag and Cu was based on the reduction of AgNO_3_ and Cu(CH_3_COO)_2_ in DMF. f-SWCNT (f-S1, 70 mg) were dispersed in 4 mL of DMF (Panreac) and magnetic stirred for 15 min. Mixtures of 35 mg of copper acetate (Cu(CH_3_COO)_2_, Alfa Aesar, MA, USA) in 2 mL of EtOH (Fischer Scientific, UK) and 35 mg of silver nitrate (AgNO_3_, from Fischer Scientific, UK) in 2 mL of EtOH were kept under magnetic stirring for 15 min and mixed with two separate suspensions of f-SWCNT in DMF. The mixtures were stirred for 78 h with an ultra-sonication step (10 min, ultrasonic bath) every 24 h. The reaction flasks were covered with aluminum foil throughout the procedure. Reaction products were filtered and washed with hexane followed by diethyl ether (Fischer Scientific, UK). Filtration and washing were carried out over a nylon membrane yielding SWCNT buckypapers.

### 2.4. Characterization of the SWCNT

#### 2.4.1. Scanning Electron Microscopy and Energy-Dispersive X-ray Spectroscopy

Scanning electron microscopy (SEM), electron backscatter diffraction (EBSD) images and EDS spectra were obtained with a Nano SEM–FEI Nova 200 (FEG/SEM) with an integrated EDAX–Pegasus X4M at high vacuum (1.0 nm at 15 kV resolution). An SUTW, Sapphire detector was used for EDS.

#### 2.4.2. Thermogravimetric Analysis

TGA measurements were performed on a TGA Q500 from T.A. Instruments^®^ (weighting precision of ± 0.01%, 0.1 µg sensitivity) at a heating rate of 10 °C/min from 40 °C to 700 °C or 800 °C, under N_2_ atmosphere, and a rate of 10 °C/min from 40 °C to 800 °C under O_2_ atmosphere, in a platinum crucible.

#### 2.4.3. X-ray Photoelectron Spectroscopy

XPS spectra were acquired with a Kratos Axis Ultra Hybrid with an Al Kα monochromated X-ray source (1486.6 eV) with a sampling depth between 3–10 nm. XPS spectra were analyzed recurring to CasaXPS software. All spectra were calibrated relative to C 1s major peak fit at 284.8 eV (C=C bond). The C 1s peak was fitted firstly with an asymmetrical peak at ~284.8 eV, attributed to C=C bonds. A wide peak at ~291 eV is attributed to ***π-π*** interactions, a non-covalent interaction of the conjugated pi-systems, in this case no FWHM nor area constraints were applied. A Gaussian function and FWHM constriction of C=C FWHM × 1 in relation to the higher intensity peak fit was used as well for other 1s peaks.

#### 2.4.4. Raman Spectroscopy

Raman spectra were obtained using a HORIBA LabRAM HR Evolution equipped with a 532 nm laser. The Raman spectrometer is coupled to a CCD detector (Horiba) and a microscope equipped with a 100× magnification objective. A grating of 1800gr/mm was used to obtain high spectral resolution. For each sample, three spectra were recorded and averaged, peak area and maximum were determined by the application of a polynomial baseline and fitting with a Lorentzian function.

Spectra in the radial breathing mode (RBM) range were acquired with a 532 nm laser with 900 gr/mm, 100% ND filter, 10 s acquisition time and four accumulations in the range of 100 to 500 cm^−1^. From the mappings two representative spectra were selected for each sample.

## 3. Results and Discussion

**Figure 1:**
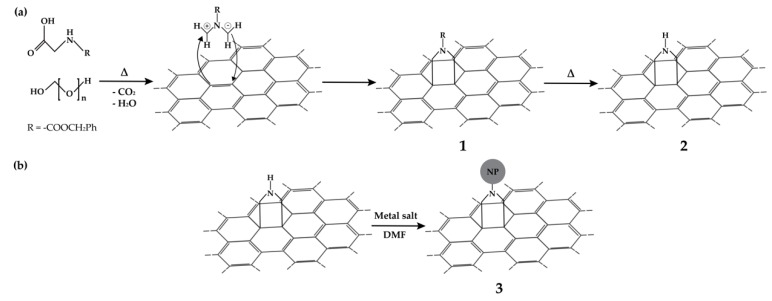
Schematics of the reactions carried out: (**a**) 1,3-dipolar cycloaddition (DCA) reaction, where 1 and 2 are the products; (**b**) anchoring of metal nanoparticles (product 3) on the pyrrolidine groups formed by the DCA reaction.

The organic functionalization studies were carried out upon SWCNT with the nanotubes purified using a method proposed in the literature [49]. The pristine and purified SWCNT were functionalized via DCA reaction, using a procedure adapted from that previously described for the functionalization of multiwall carbon nanotubes [29]. The reaction scheme is presented in Figure 1, showing the formation of the reactive dipole by heating paraformaldehyde and Z-Gly-OH, and its reaction products on the SWCNT wall. The DCA reaction was carried out varying the Z-Gly-OH content and the reaction temperature, and the functionalization yield was evaluated by XPS analysis. Finally, the decoration of the f-SWCNT with metal NP via reduction of the metal salts in DMF and anchoring on the nanotubes was carried out.

### 3.1. Scanning Electron Microscopy

**Figure 2:**
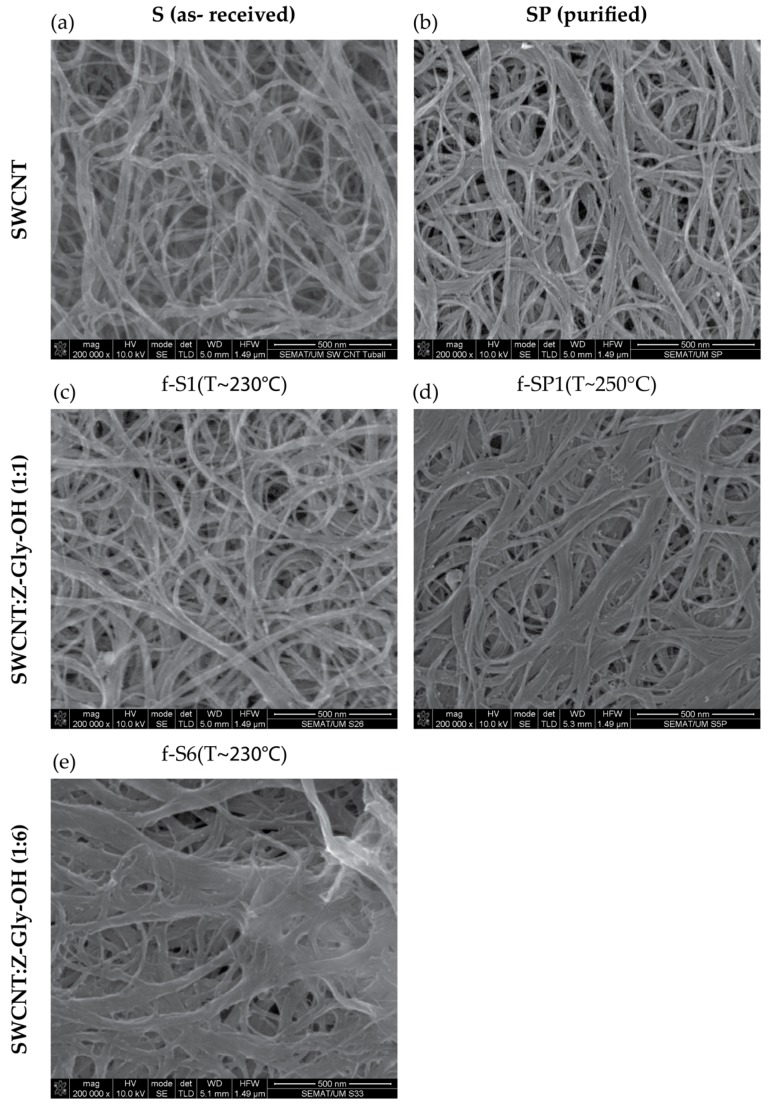
SEM micrographs of the (**a**) pristine, (**b**) purified, and (**c**–**e**) functionalized SWCNT.

Figure 2 illustrates the morphology of the SWCNT after each step of chemical modification. The SEM images show the structural integrity of the SWCNT after purification and functionalization. 

**Figure 3:**
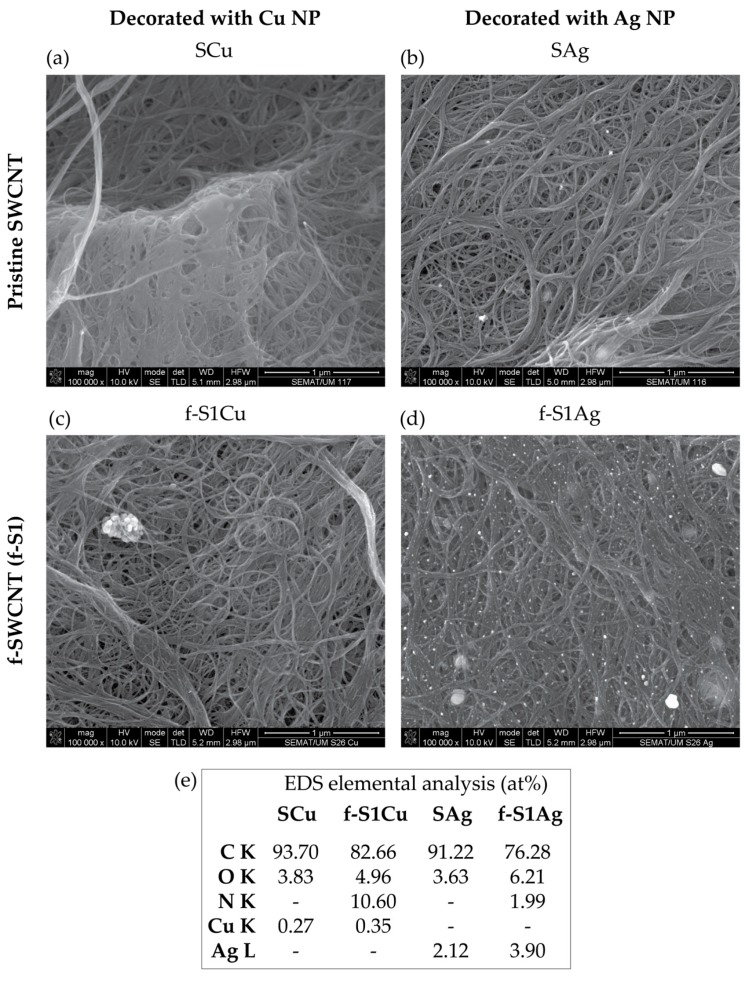
SEM images of the SWCNT, (**a,b**) pristine and (**c,d**) functionalized, after reduction of metal NP in dimethylformamide (DMF), and (**e**) corresponding energy dispersive spectroscopy (EDS) results.

Figure 3 illustrates the higher yield of NP formation and anchoring on the f-SWCNT relative to the same procedure performed on pristine SWCNT. This was particularly clear for Ag NP whereas Cu NP were observed to form large clusters (albeit nano-sized). The EDS spectra demonstrates the presence of nitrogen-containing groups as well as Cu and Ag in metal form. EDS is a local surface measurement that may be used in combination with SEM to estimate the metal NP size. However, the measurements are difficult to perform due to the small size of the metal nanoparticles and the interference of the Fe catalyst. Images highlighting the distribution of Fe and Ag separately are shown in the supplementary materials (Figure S1b,c). Quantitative analysis of the overall Fe, Ag, and Cu contents were performed for the functionalized and decorated samples using TGA.

### 3.2. Functionalization of the SWCNT

**Figure 4:**
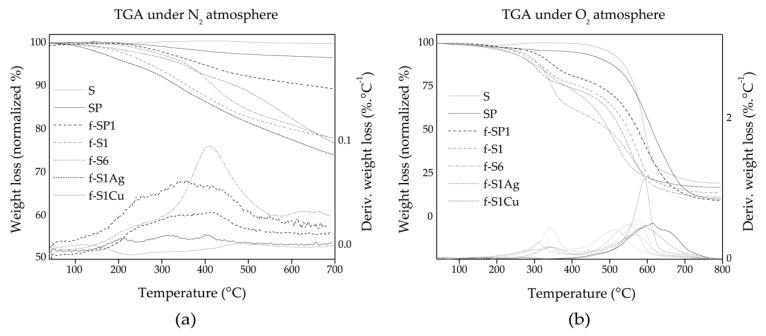
Normalized thermogravimetric analysis (TGA) curves and corresponding derivative: (**a**) under N_2_ atmosphere for pristine, purified and functionalized SWCNT (f-SWCNT); (**b**) pristine, purified, f-SWCNT, and hybrid SWCNT (h-SWCNT), under O_2_ atmosphere.

SWCNT may be heated to temperatures above 2000 °C under inert atmosphere without significant weight loss and keeping their structural integrity. Thus, the weight loss observed during TGA testing under inert atmosphere may be attributed to loss of the organic functionalization of the SWCNT. Organic compounds typically decompose above 180–200 °C yielding volatile products and thus undergoing weight loss. Figure 4a shows normalized TGA curves for the SWCNT and f-SWCNT under an N_2_ atmosphere, showing mass loss up to 21.85%. Thermal decomposition occurs through at least two degradation steps starting above 150 °C and extending up to 500 °C. Considering the scheme in Figure 1 the major degradation steps may be assigned to cleavage of the R group in 1 and decomposition of the remaining pyrrolidine group in 2. Comparing the TGA curves for f-S1 and f-SP1 (the SWCNT that were functionalized with similar reagent ratios but different reaction temperature) a larger overall weight loss is observed for f-S1 indicating that, if similar functionalization yield was achieved, f-S1 is functionalized with a larger fraction of >NR (product 1 in Figure 1) compared to f-SP1. Table 2 shows that the overall weight loss was much lower for f-SP1 compared to the other f-SWCNT, which is consistent with a larger contribution of the “lighter” group 2 in f-SP1. f-S6 was produced using a large excess of amino acid, showing what appears to be a large organic layer by SEM, however presenting an overall weight loss similar to that of f-S1. The major degradation step occurs near 400 °C which is consistent with the presence of a stable pyrrolidine group.

Heating under O_2_ all organic carbon undergoes burn-off below 600 °C, leaving only inorganic matter and metals/oxidized metals. For SWCNT and functionalized SWCNT, the residue formed corresponds mostly to iron oxide for pristine, purified, and f-SWCNT, and iron, silver, or copper oxides for h-SWCNT. The oxidative degradation TGA curves for pristine SWCNT (S), f-SWCNT (f-S1) and corresponding h-SWCNT (f-S1Ag and f-S1Cu) is shown in Figure 4b. Thermal-oxidative degradation initiates above 250 °C for all functionalized materials showing the larger degradation step above 500 °C. Pristine SWCNT are characterized by an incipient weight gain above 200 °C due to oxidative processes, and a steep decomposition step near 500 °C. The temperature of maximum degradation rate of SP (peak temperature for the first derivative of the TGA curve), listed in Table 2, is shifted to higher temperature by approximately 20 °C as compared to as-received SWCNT (S), confirming the effectiveness of the purification process. The f-SWCNT show in general a weight loss starting at lower temperature due to the thermal degradation of the organic groups bonded to their sidewalls.

The Fe content was estimated from the weight % residue obtained under O_2_ (Table 2). The results show that the functionalization itself induces a decrease of the catalyst content, of the same magnitude as the purification step (~4 wt.% lower than pristine SWCNT).

**Figure 5:**
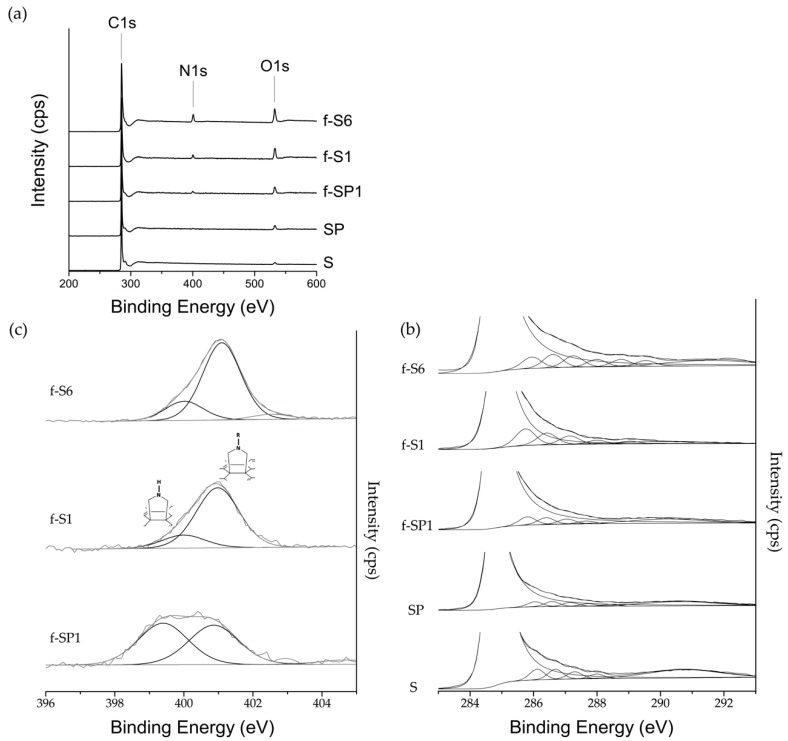
(**a**) Wide scan X-ray photoelectron spectroscopy (XPS) spectra of pristine, purified, and f-SWCNT; deconvolution of high resolution XPS spectra of (**b**) C 1s peak of f-SWCNT, purified, and pristine SWCNT; and (**c**) N 1s of f-SWCNT.

**Figure 6:**
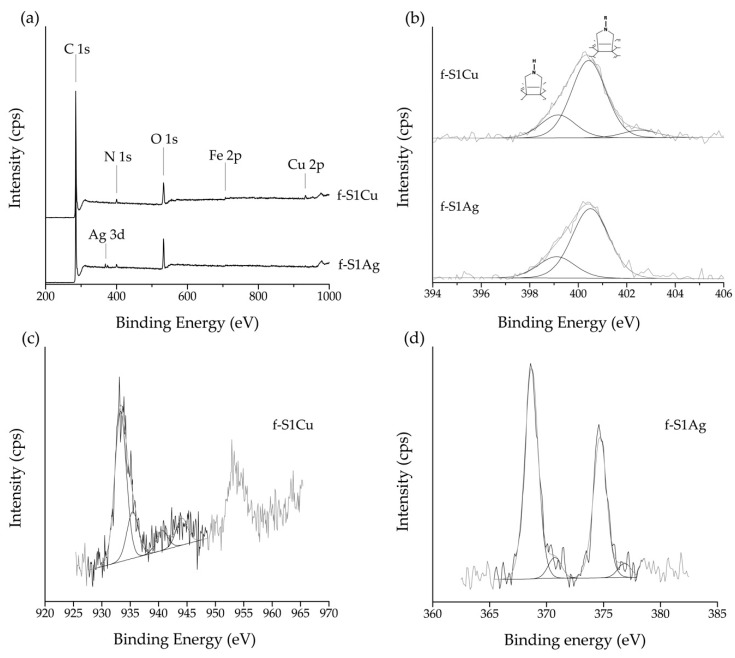
(**a**) Wide scan XPS spectra of h-SWCNT; deconvolution of high resolution XPS spectra of h-SWCNT (**b**) N 1s peak; (**c**) Cu 2p peak of f-S1Cu; and (**d**) Ag 3d peak of f-S1Ag.

Wide scan XPS spectra of pristine, purified, f-SWCNT, and h-SWCNT are shown in Figure 5a. The N 1s peak is present for all the f-SWCNT and h-SWCNT. A considerable increase of the O 1s peak intensity is observed relative to pristine SWCNT, increasing in the order: S < SP < f-SP1 < f-S1 < f-S6. The presence of Cu and Ag is confirmed by the peaks centered at 933.40 eV and 368.49 eV, respectively, for f-S1Cu and f-S1Ag, as shown in Figure 6.

The high-resolution C 1s spectra depicted in Figure 5b for the untreated, purified, and functionalized SWCNT are characterized by a main component peak centered at 284.8 eV, assigned to the sp^2^ C of the graphene-like SWCNT lattice, and a strong tailing towards the higher binding energy side. This tailing is due to the conjugation of the π-electrons in the polyaromatic SWCNT lattice, and complicates the C 1s peak analysis. The deconvolution of the C 1s spectra show the absence of component peaks above 288 eV for S, SP, and f-SP1 nanotubes, while f-S1 and f-S6 show peaks at 288 eV and above. The lower binding energy (BE) range 285–287.5 eV may be assigned to C–O and C–N bonds (Ph–OH, Ph–CH_2_–O–, C–N–C, for example) while carbonyl in functional groups such as ester or amide (O=C–O–, O=C–NH) are associated to higher BE, above 288 eV. Thus, pristine and purified SWCNT, as well as f-SP1 contain a sPLPmall contribution of carbonyl functional groups, while the surface composition of f-S1 and f-S6 contain a considerable concentration of this class of functional groups. Atomic percentages calculated for the peak components, as well as additional figures, can be found in the supplementary materials (Table S1, Figures S2 and S3). The deconvolution of the high-resolution N 1s spectra yields two peaks centered at 400.5 eV (corresponding to >N–COO–, as in benzyl carbamate) and 399.2 eV (corresponding to >NH, as in pyrrolidine) as represented in Figure 5c. The ratio of >NH: >N–COO– measured for the f- and h-SWCNT is listed in Table 3, showing that f-SP1, functionalized at higher temperature, contains equal composition of benzyl carbamate (1) and pyrrolidine (2) while the remaining f- and h-SWCNT, functionalized at lower temperature, present approximately 20% of the functional groups in pyrrolidine form. Table 4 shows that the N at% is relatively similar for SP5, f-S1, f-S1Ag, and f-S1Cu, presenting similar N:C ratios (Table 3). Since the DCA reaction is the only source of N, the N at% is a good indicator of the degree of organic functionalization reached. Conversely, the measured surface oxygen originates from different sources: the pristine and purified SWCNT contain oxygen, functionalized SWCNT contain oxygen associated to the O=C–NH in benzyl carbamate, and under oxidizing conditions, f-S1Ag and f-S1Cu contain oxygen compounds of inorganic origin (Cu or Ag oxides, traces of Cu or Ag salts), thus complicating the estimate of oxygen resulting from the DCA reaction alone.

Evidence for the presence of Ag and Cu on h-SWCNT is provided by the wide scan XPS spectra (Figure 6a). High resolution spectra of N 1s for f-S1Ag and f-S1Cu are similar to those observed for f-S1 (Figure 6b), however slightly shifted to lower eV. The Ag 3d high resolution XPS spectrum of f-S1Ag (Figure 6d) shows the characteristic peaks of the Ag metallic form (BE 368.6 eV for 3d_5/2_ and 374.6 ± 0.07 eV for 3d_3/2_ [54]) thus indicating that the reduction of the Ag salt was effective. Nanosized Ag dots are observed at the surface of the pristine SWCNT and, at much higher concentration, on the surface of the f-SWCNT (Figure 3 and Figure S1). High resolution spectra of Cu 2p presents an asymmetric 2p_3/2_ peak near 933.3 eV which may be assigned to Cu(I).species, indicating a partial reduction of Cu(CH_3_COOH)_2_ [54,55].

### 3.3. Single Walled Carbon Nanotube Structure and Functionalization

Raman spectroscopy is a non-destructive technique that analyses the inelastic scattering of light and has been widely used to characterize graphitic materials. The Raman spectrum of SWCNT is characterized by two first-order modes, the G band at ~1580 cm^−1^, split into several shoulders, and the RBM in the frequency range of 100–500 cm^−1^, with frequency that is inversely proportional to the tube diameter [53]. The Raman mode D (~1350 cm^−1^) is sensitive to defects in the sp^2^ carbon lattice while 2D band (~2670 cm^−1^), with two times the frequency of D band, results from resonance effects [56].

**Figure 7:**
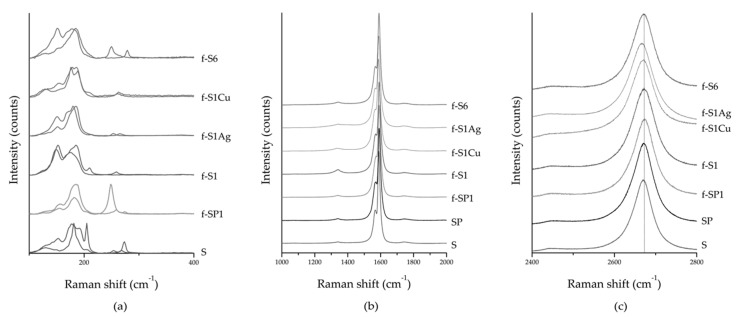
(**a**) radial breathing mode (RBM), (**b**) D, G, and (**c**) 2D Raman peaks of pristine, purified, f-SWCNT, and h-SWCNT.

Raman spectra of pristine, purified, f-SWCNT, and h-SWCNT are presented in Figure 7. The RBM region depicted in Figure 7a shows changes in frequency induced by chemical functionalization. These changes are observed across the whole RBM frequency range with no clear evidence for preferential functionalization of SWCNT with specific chirality. The RBM modes continue to be active for the functionalized SWCNT, they do not disappear as reported for oxidized SWCNT [57] denoting that the nanotube structure was not damaged. Additional data included as supplementary material allowed a rough estimate of the SWCNT diameter, showing a similar diameter range for as received and functionalized SWCNT (Figure S4 and Table S2). Figure 7b presents the D and G bands of f-SWCNT and h-SWCNT illustrating their similarity to the pristine and purified SWCNT in terms of intensity and area. A small increase in the intensity of the D band is observed for samples f-S6 and f-S1, confirming that the functionalization did not induce extensive damage. A downshift of ~4 cm^−1^ is observed for the 2D band for both types of h-SWCNT (Figure 7c) which may be induced by charge transfer between SWCNT and the metal anchored at the SWCNT surface [58].

## 4. Conclusions

A simple one-pot and solvent-free method based on the 1,3-dipolar cycloaddition reaction of azomethine ylides was implemented for the functionalization of SWCNT. The reaction yield, in terms of functional groups bonded to the SWCNT, was observed to vary with reagent concentration, while an increase in the reaction temperature resulted in further cleavage of the protecting group in the pyrrolidine nitrogen without decrease of functional group concentration.

XPS analysis showed that the reduction of AgNO_3_ in the presence of SWCNT and f-SWCNT in DMF was effective, yielding metallic Ag NP at the nanotube surface. A similar procedure was tested for the reduction of Cu(CH_3_COO)_2_ however, under the reaction conditions tested, only partial reduction was achieved for the organic Cu salt. A higher yield of Ag NP was observed for the f-SWCNT compared to the pristine SWCNT, indicating that the functionalization plays a role in the formation and stabilization of Ag NP at the SWCNT surface. Raman spectroscopy demonstrated the structural quality of the SWCNT before and after functionalization, while showing changes in the RBM region that may be related to functionalization selectivity and may be interesting to study further. The 2D band of the h-SWCNT presented a small downshift relative to SWCNT and f-SWCNT that may relate to charge transfer with the NP anchored at the SWCNT surface.

## Figures and Tables

**Table 1:** Compositions of single walled carbon nanotubes (SWCNT), N-benzyloxycarbonylglycine (Z-Gly-OH), and paraformaldehyde (PFA) used for the 1,3-dipolar cycloaddition reaction.

Sample	SWCNT	SWCNT:Z-Gly-OH	Z-Gly-OH:PFA	Temperature (°C)	Reaction Time (h)
f-S1 ^1^	Pristine	1:1 (wt)	1:6 (mol)	230 ± 10	2
f-S6	Pristine	1:6 (wt)		230 ± 15	
f-SP1	Purified	1:1 (wt)		250 ± 13	

^1^ Sample used for the decoration with metal nanoparticles (NP).

**Table 2:** TGA results for the analysis performed under nitrogen and under oxygen atmosphere.

	**In Nitrogen**			
**Sample**	**1st Derivative peak T (°C)**		**Weight loss (wt.%) @700 °C**	Approx. residue from Cu or Ag anchoring
	T1	T2		
S	-	-	0.19	-
SP	-	-	5.09	-
f-S6	200	409	22.04	-
f-SP1	240	345	14.74	-
f-S1	175	335	21.72	-
f-S1Cu	203	367	16.95	4.8
f-S1Ag	170	331	15.06	6.7
	**In Oxygen**			
	1st Derivative peak T (°C)		Fe (wt.%)	Residual weight (wt.%) @800 °C
	T1	T2		
S	-	592	13.7	19.64
SP	269	614	10.9	15.53
f-S6	341	572	8.3	11.81
f-SP1	344	593	6.7	9.64
f-S1	338	578	10.0	14.35
f-S1Cu	311	552	~10.0	17.5
f-S1Ag	337	515	~10.0	17.7

**Table 3:** Fraction of N at% relative to C and O, and the ratio >NH: >NR (pyrrolidine group to benzyl carbamate group) as measured by XPS.

	N:C	N:Oorg	>NH:>NR
**f-SP1**	0.02	2.36	1.05
**f-S1**	0.02	0.43	0.17
**f-S1Ag**	0.02	0.34	0.19
**f-S1Cu**	0.02	0.28	0.25
**f-S6**	0.05	0.89	0.25

**Table 4:** Atomic percentages of the elements present in each sample, as obtained by XPS.

	Atomic %					
	C 1s	N 1s	(O 1s)_org_^1^	Ag 3d	Cu 2p	Fe 2p
**S**	97.79	-	1.30	-	-	0.64
**SP**	96.55	-	3.2	-	-	0.25
**f-SP1**	94.78	1.57	0.67	-	-	-
**f-S1**	91.2	2.13	4.93	-	-	0.12
**f-S1Ag**	90.36	1.67	4.90	0.09	-	0.11
**f-S1Cu**	92.03	1.4	5.09	-	0.08	0.13
**f-S6**	88.32	4.52	5.08	-	-	0.58

^1^ Atomic % corrected accounting for the oxygen bonded to traces of Si.

## Supplementary Materials

The following are available online at https://www.mdpi.com/2079-4991/9/10/1416/s1, Figure S1: **(a)** SEM image of single walled carbon nanotubes (SWCNT) f-S1_Ag; images of the distribution of **(b)** Fe nanoparticles; and **(c)** Ag nanoparticles, as obtained by energy dispersive spectroscopy (EDS) mapping, Table S1: Atomic percentages of the deconvoluted peaks of C 1s high resolution X-ray photoelectron spectroscopy (XPS) spectra, Figure S2: High resolution XPS spectra of C 1s peaks of (**a**) purified samples before and after functionalization and as-received SWCNT; (**b**) non-purified samples of functionalized and as-received SWCNT, Figure S3: Deconvolution of high resolution XPS spectra of C 1s peaks of the amine-functionalized SWCNT, Figure S4: Radial breathing mode (RBM) peaks for the amine functionalized and as-received SWCNT at laser energy of **(a)** 2.33 eV; **(b)** 1.96 eV; and **(c)** 1.58 eV, Table S2: Estimated diameters, at different laser excitation, for as-received and functionalized SWCNT.
