# Supplementary material for: A Simple Method for Anchoring Silver and Copper Nanoparticles on Single Wall Carbon Nanotubes
Source: Nanomaterials (Basel). 2019 Oct 4;9(10):1416. doi: 10.3390/nano9101416 (PMC6835420; doi:10.3390/nano9101416)
Supplement: Supplementary file 1 [file nanomaterials-09-01416-s001.pdf]

nanomaterials

MDPI

Article

A Simple Method for Anchoring Silver and Copper Nanoparticles on Single Wall Carbon Nanotubes

Mariana M. Silva [1], Daniel Ribeiro [1], Eunice Cunha [2], M. Fernanda Proença [3], Robert J. Young [2] and Maria C. Paiva [1,*]

[1] Institute for Polymers and Composites, University of Minho - Campus of Azurém, 4800-058 Guimarães, Portugal; b8523@dep.uminho.pt (M.M.S.); daniel.ribeiro@dep.uminho.pt (D.R.)
[2] National Graphene Institute and School of Materials, University of Manchester, Manchester M13 9PL UK; eunice.cunha-2@manchester.ac.uk (E.C.); robert.young@manchester.ac.uk (R.J.Y.)
[3] Centre of Chemistry, University of Minho - Campus of Gualtar, 4710-057 Braga, Portugal; fproenca@quimica.uminho.pt
* Correspondence: mcpaiva@dep.uminho.pt

Received: 11 September 2019; Accepted: 1 October 2019; Published: date

Supplementary Material

1. Observation of Ag nanoparticles by SEM/EDS

The measurement of the Ag nanoparticles size using SEM and energy dispersive spectroscopy (EDS) is difficult to perform due to the image resolution, the small size of the metal nanoparticles, and the interference of the Fe catalyst. The presence of metal nanoparticles could be observed by SEM/EDS, as depicted in Figure S1 (a). Fe and Ag nanoparticles could be observed separately by EDS mapping and image treatment. The images for Fe and Ag EDS mappings (Figure S1 (b) and (c), respectively) illustrate the presence of both types of nanoparticles on hybrid functionalized SWCNT.

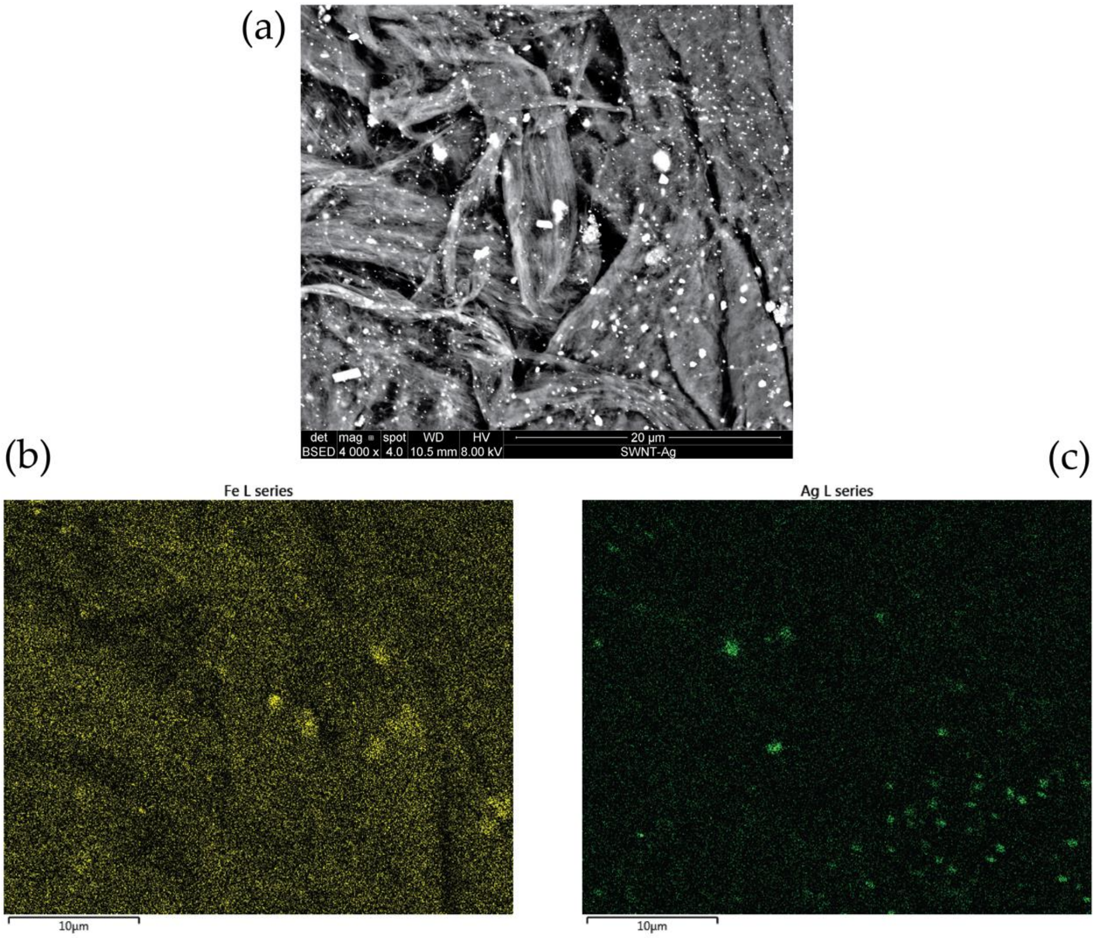

Figure S1. (a) SEM image of single walled carbon nanotubes (SWCNT) f-S1_Ag; images of the distribution of **(b)** Fe nanoparticles; and **(c)** Ag nanoparticles, as obtained by energy dispersive spectroscopy (EDS) mapping.

2. X-Ray photoelectron spectroscopy

Table S1 presents the atomic percentages, calculated by deconvolution of the high-resolution C 1s peak. The binding energies of the deconvoluted C 1s peak components were attributed according to the electronegativity of the atoms, the higher the electronegativity of the bonded atoms the lower the binding energy.

Table S1. Atomic percentages of the deconvoluted peaks of C 1s high resolution X-ray photoelectron spectroscopy (XPS) spectra.

	Atomic percentages of C bonds (%)		
Binding Energy (eV)	284.8	285 – 288	> 288
Assigned Bonds	C=C	C-C<br>C-N<br>C-O	O=C-O-<br>O=C-N
S	92.5	7.5	0
SP	93.3	6.9	0.8

f-SP1	92.9	6.5	0.6
f-S1	79.6	17.3	3.1
f-S6	84.2	10.1	5.7
f-S1_Ag	55.5	43.0	1.5
f-S1_Cu	73.7	24.8	1.6

Normalized high resolution C 1s peaks of the materials are shown in Figure S2. Wider C 1s peaks are observed for all the treated single walled carbon nanotubes (SWCNT) relative to the as received SWCNT. The chemical groups resulting from functionalization are assigned to the 285–288 eV range. The functionalized SWCNT that were not purified previous to functionalization present a higher contribution of carbonyl containing groups (Figure S2 (b)). Figure S3 shows the deconvolution of C 1s high resolution peaks of the organically functionalized samples and the corresponding attributed bonds.

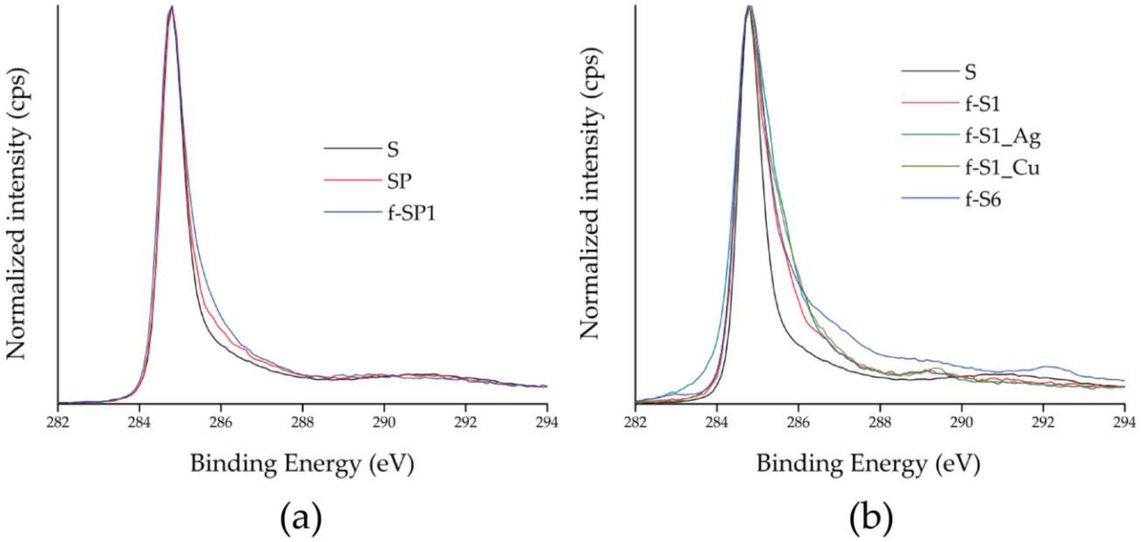

Figure S2. High resolution XPS spectra of C 1s peaks of (**a**) purified samples before and after functionalization and as-received SWCNT; (**b**) non-purified samples of functionalized and as-received SWCNT.

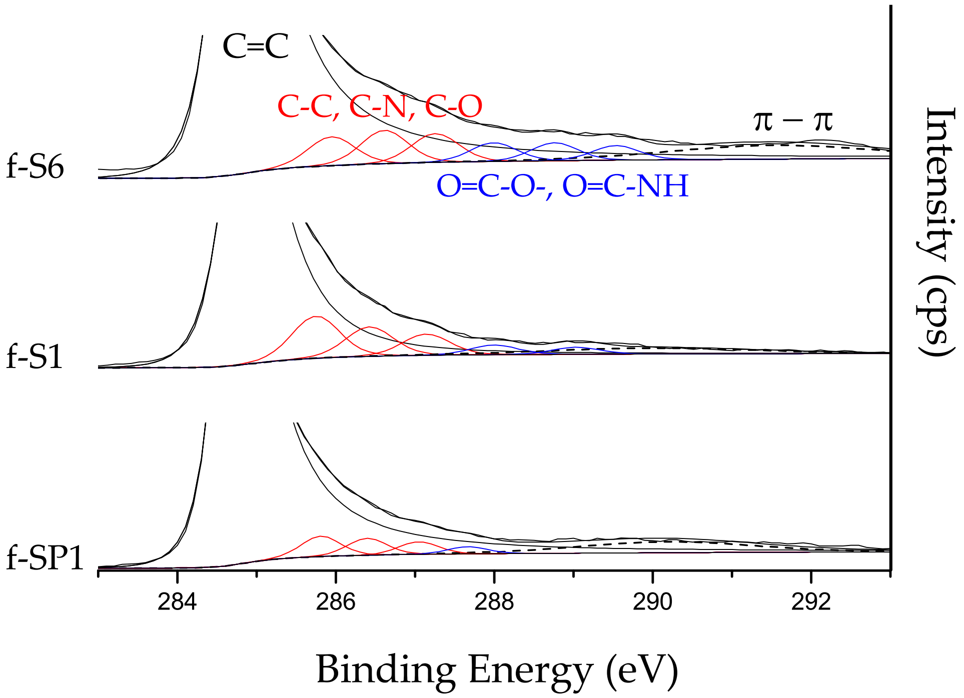

Figure S3. Deconvolution of high resolution XPS spectra of C 1s peaks of the amine-functionalized SWCNT.

3. Raman spectroscopy

Recorded Raman spectra show that the SWCNT morphology may vary from bundles to bundle [1]. This is illustrated in Figure S4 (a), where two spectra acquired in different areas for each type of functionalized SWCNT is presented, showing different peaks, for spectra recorded with a laser of 532nm. The spectra obtained also varies with the laser excitation energy, showing shifts in the dominant peaks (Figure S4 (b) and (c)).

Radial breathing mode (RBM) originate on the bond-stretching out-of-plane phonon mode for which C atoms move in the radial direction, allowing the determination of the diameter of tubes (dt) by using Equation (S1) [2],

$$w = \frac{C_1}{d_t} + C_2 \qquad \text{Equation (S1)}$$

where C_1 and C_2 are constants estimated experimentally, w is the laser energy and d_t the tube diameter. The constants C_1 = 218.7 nm.cm^{-1} and C_2 = 15.3 cm^{-1} [3] where used to estimate the diameters of the SWCNT in the bundles after a Lorentzian fit to the RBM lines. The diameters estimated for laser excitation of 532 nm, 633 nm, and 785 nm are presented in Table S2.

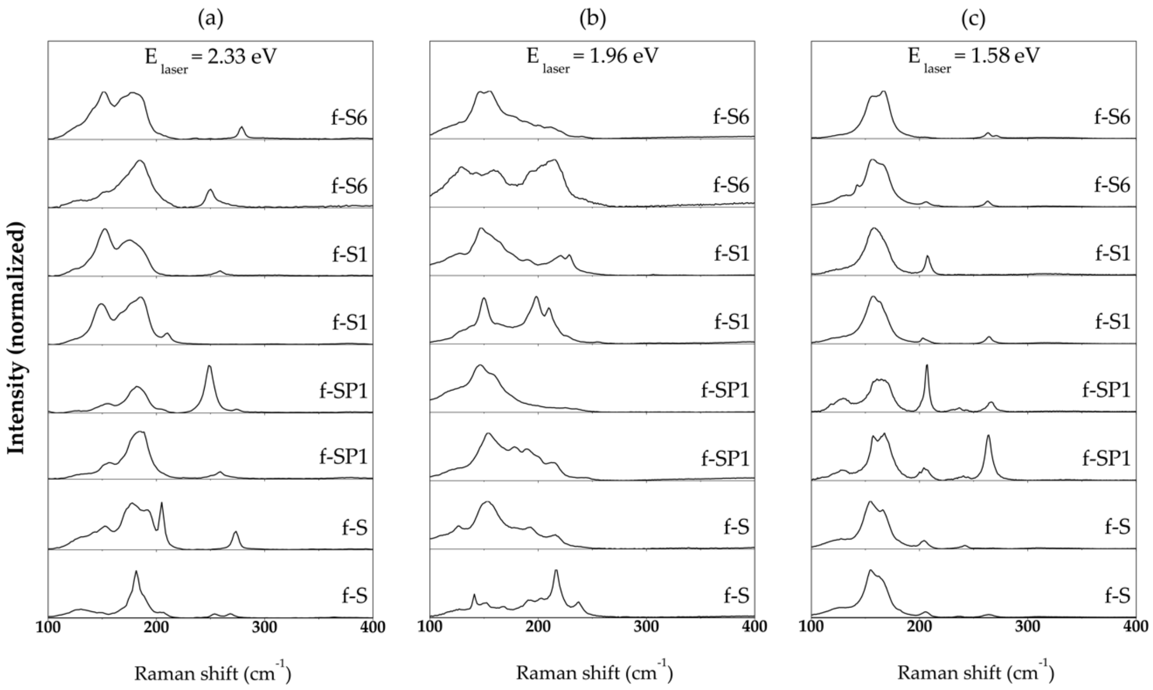

Figure S4. Radial breathing mode (RBM) peaks for the amine functionalized and as-received SWCNT at laser energy of **(a)** 2.33 eV; **(b)** 1.96 eV; and **(c)** 1.58 eV.

Table S2. Estimated diameters, at different laser excitation, for as-received and functionalized SWCNT.

Laser	Diameter (nm)			
	**As-received**	**f-SP1**	**f-S1**	**f-S6**
**1.58 eV**	2.02	2.02	1.96	1.92
	1.57	1.58	1.55	1.72
	1.45	1.45	1.45	1.56
	1.16	1.17	1.17	1.44
	0.98	0.97	1.15	0.89
	0.89	0.89	0.89	0.86
**1.96 eV**			2.02	
	1.97		1.80	
	1.74	1.68	1.66	
	1.62	1.59	1.63	
	1.44	1.54	1.51	1.91
	1.25	1.35	1.44	1.69
	1.17	1.26	1.20	1.55
	1.10	1.11	1.13	
	1.00		1.08	
			1.03	

**2.33 eV**	1.89			
	1.60	1.57	1.64	1.76
	1.32	1.34	1.60	1.61
	1.27	1.27	1.39	1.40
	1.24	0.95	1.35	1.29
	1.16	0.91	1.28	0.94
	0.93	0.85	1.13	0.84
	0.86			

1. Yu, Z.H. and L. Brus, Rayleigh and Raman scattering from individual carbon nanotube bundles. Journal of Physical Chemistry B, 2001. **105**(6): p. 1123-1134.

2. Hodge, S.A., et al., Unweaving the rainbow: a review of the relationship between single-walled carbon nanotube molecular structures and their chemical reactivity. Chemical Society Reviews, 2012. **41**(12): p. 4409-4429.

3. Fogden, S., et al., Scalable method for the reductive dissolution, purification, and separation of single-walled carbon nanotubes. ACS nano, 2011. **6**(1): p. 54-62.
